# Quantifying the Income-Increasing Effect of Digital Agriculture: Take the New Agricultural Tools of Smartphone as an Example

**DOI:** 10.3390/ijerph20043127

**Published:** 2023-02-10

**Authors:** Xin Luo, Shubin Zhu, Zhenjiang Song

**Affiliations:** 1 College of Economics and Management, Jiangxi Agricultural University, Nanchang 330045, China; 2 Rural Development Research Center of Jiangxi Province, Jiangxi Agricultural University, Nanchang 330045, China; 3 Institute of Jiangxi Selenium-Rich Agricultural Research, Jiangxi Agricultural University, Nanchang 330045, China

**Keywords:** digital agriculture, new farming tools for smartphones, income-generating effects, instrumental variables approach, mediating effects

## Abstract

Smartphones are increasingly used in rural areas and have become indispensable new farming tools in farmers’ production and their lives. Based on data from the 2018 China Household Tracking Survey, this study uses ordinary least squares regression with two-stage least squares as a benchmark regression to investigate the impact of the extent of smartphone use on farm household income. Our findings are as follows. ① The degree of use of new smartphone farming tools has a significant income-increasing effect on farm households. ② There is variability in the impact of the use of new smartphone farming tools on the income of farmers in different regions. The highest income-generating effects on the use of smartphone tools were found in the western region, followed by the eastern region, with the smallest effects found in the central region. ③ Low-income farmers have the highest income effects from using new smartphone farming tools. We therefore recommend further improving the digital infrastructure in rural areas to give full play to the driving force of digital technology.

## 1. Introduction

Since the 20th century, the new round of industrial revolution based on digital technology has swept the globe. The new economic model characterized by the digital economy has risen rapidly, changing the structure of global wealth. At present, China’s total economic output has jumped significantly, and residents’ incomes have increased year after year, but the trickle-down effect of economic growth has failed to cover all groups, and the income gap is slightly on the rise. According to the China Digital Economy Development Report (2022), the size of China’s digital economy reached 45.5 trillion yuan, with the total digital economy accounting for 39.8% of GDP. By December 2020, the number of Internet users in rural areas of China had reached 309 million, and the proportion of villages connected to optical fiber as well as 4G networks exceeded 98%. Since the reform and opening up, China has experienced 40 years of rapid growth [1]. With the comprehensive victory in the battle against poverty and the total elimination of absolute poverty [2], the trend of slower growth in farmers’ income has become more and more pronounced. According to the National Bureau of Statistics, the real growth rates of rural residents’ disposable income in China from 2017 to 2020 were 7.3%, 6.6%, 5.8%, and 3.8%, respectively, a significant slowdown.

The realistic background releases two signals. Firstly, there is a mismatch between the traditional agricultural economic development model and the modern digital development context [3]. The Chinese government is vigorously promoting the construction of new 5G infrastructures, effectively promoting new paths for digital agriculture development, from which the traditional smallholder production model can hardly reap policy dividends. Secondly, the income gap between farming households has widened. Data shows that, as of 2018, the income of the highest 20% of rural residents with per capita disposable income was 9.29 times higher than that of the lowest 20% of rural residents. This wide income gap makes it difficult to create sustained high-income growth and poses certain problems for rural governance and social stability [4]. Solving the problem of insufficient farmer income growth and discovering new momentum for farmer income growth are urgent challenges for China.

The income-generating effects of digital agriculture development manifest four important phenomena. First, digital agriculture development is conducive to expanding agricultural production capacity [5], thus forming a production scale effect [6]. Digital agricultural operations can help promote the precision of intelligent agriculture [7]. They also help farmers allocate agricultural production resources and carry out scientific planting and production [8]. Digital farming can help promote the precise allocation of agricultural production resources and scientific cultivation. It also enables smart farming to create a good management and production basis for large-scale planting, and access to the Internet of Things allows agricultural production to produce an intensive effect [9].

Secondly, digital agriculture unblocks the circulation of agricultural products and broadens sales paths. The development of digital agriculture can effectively promote the problem of asymmetric market information [10] while also effectively mitigating market information asymmetry, promoting the transparency of the whole industrial chain of agricultural products, and ensuring the stable production of high-quality, well-priced agricultural goods [11]. In addition, the transformation of digital agriculture brings about resource-sharing between producers and consumers of agricultural products, effectively promoting in-depth interactions between them [12]. Digital agriculture makes the intelligent circulation of agricultural products through e-commerce platforms a reality, breaking the time and space boundaries of traditional market transactions [13]. Third, the development of digital agriculture is conducive to improving the human capital of farmers and increasing their employment opportunities [14]. Farmers can effectively gain more employment, education, and training possibilities [15]. Fourth, with the impact of digital agriculture on traditional agriculture, the integration of consumer markets and value chains has brought about the integration of the tertiary sector, represented by cultural tourism and agriculture [16]. This gives digital agriculture an educational and social function and provides more employment possibilities for farmers.

The continuous integration of the digital economy and the real economy has increased total factor productivity, and digital agriculture, with the integration of the Internet industry and the agricultural industry, has become an important element in the development of the new real economy of agriculture [17]. The smartphone, the main Internet terminal, is currently the most widely covered Internet access device for farmers in the world because of its portability and relatively low threshold of use [18]. China is particularly well-positioned for mobile internet development. It has a particularly good foundation for mobile internet construction, with the number of rural internet users in China reaching 255 million in 2020, accounting for 28.2% of global Internet users. The 2021 Central Government No. 1 document on accelerating the modernization of agriculture and rural areas by comprehensively promoting the revitalization of the countryside states, “Implement digital countryside construction and development projects, promote rural gigabit optical networks, fifth generation mobile communications (5G), and mobile Internet of Things (IoT) to be planned and built in tandem with cities.” The support of a series of policies has promoted the expansion of Internet infrastructure in rural areas and laid the foundation for the development of mobile Internet in rural areas. Smartphones, which are an integral part of digital agriculture, have favorably promoted the development of digital agriculture and have become a new farming tool for farmers in the new era.

In summary, existing studies have discussed the income-generating effects of digital agriculture in promoting the large-scale production of agricultural products and by promoting networked marketing, facilitating industrial integration, and improving the human capital of farmers. However, there is a lack of comparative analysis on the impact of different levels of smartphone use on the income of farmers. Most of the relevant studies have been conducted from a macro perspective, and there is a lack of empirical evidence on the micro-level impacts of smartphone use on farmers’ incomes—which is not in line with the various agricultural informatization policies currently in place in China. This may be due to the fact that the studies focus on developing countries with poor information technology bases and low levels of rural information and communication technology (ICT) [19]. However, due to the relatively high Internet penetration and mobile Internet usage in rural areas of China [20], farmers’ ability to use new farming tools on their smartphones can have deeper impacts on farm incomes than simple smartphone use. Drawing on the EU’s Digital Competence Framework and UNESCO’s Global Framework for Digital Literacy, this paper examines the impact of new smartphone farming tools on farmers’ income [21]. In order to reflect real-life scenarios, this study divides the use of new smartphone farming tools into four categories: basic use, work-learning, digital life, and social.

As rural informatization continues to advance, smartphones, mobile network terminals that are portable, easy to use, and relatively inexpensive, are better able to meet farmers’ needs than, for example, computers [22]. This paper first examines the relationship between digital agriculture and new smartphone farming tools. It begins by analyzing the intrinsic link between digital agriculture and new farming tools for smartphones, sorting out the current main paths to increase income from digital agriculture, and putting forward relevant hypotheses. The paper then systematically conceptualizes the mechanisms through which new smartphone farming tools can increase income in the context of China’s new 5G infrastructure. It also conducts an econometric analysis through ordinary least squares regression (OLS), the instrumental variable method, mediating effects, stratified regression, and grouping regression. From these, this study draws some conclusions regarding a feasible strategy for promoting digital agriculture in terms of farmers’ income-generating effects in a targeted manner to provide useful references for the development of digital agriculture in China.

## 2. Theoretical Analysis

**Figure 1:**
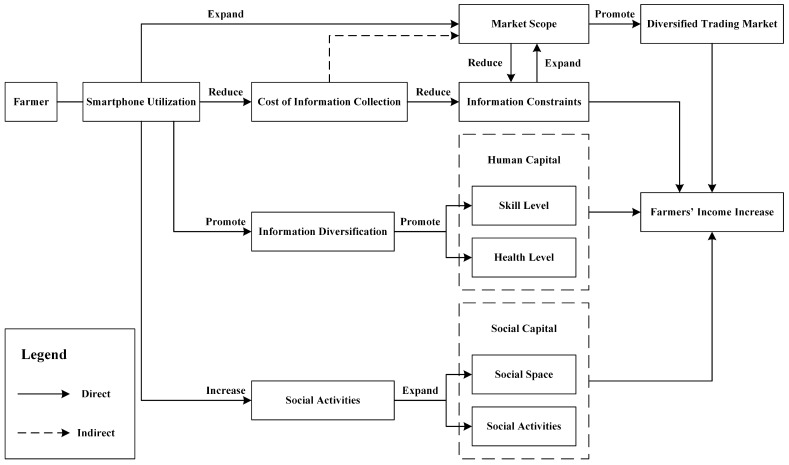
Mechanisms of the impact of new smartphone farming tools on farm household income. Note: The mechanisms have pointed three theoretical path. First, *Smartphone Utillization of Farmer* ⟶ (*Expand*) ⟶ *Market Scope* ⟶ (*Promote*) ⟶ *Diversified Trading Market*, while *Smartphone Utillization of Farmer* ⟶ (*Reduce*) ⟶ *Cost of Information Collection* ⟶ (*Reduce*) ⟶ *Information Constraints*. The ultimate goal of these paths is *Farmer’s Income Increase*, which shows that the use of new farming tools for smartphones will have an income-generating positive effect on farmers. Second, *Smartphone Utillization of Farmer* ⟶ (*Promote*) ⟶ *Information Diversification* ⟶ (*Promote*) ⟶ *Human Capital* ⟶ *Farmer’s Income Increase*, which shows that the use of new farming tools for smartphones will have different income-generating effects for different income groups. Third, *Smartphone Utillization of Farmer* ⟶ (*Increase*) ⟶ Social Activities ⟶ (*Expand*) ⟶ Social Capital ⟶ *Farmer’s Income Increase*, which shows that the use of new smartphone farming tools will have a different income income-generating effect on farmers in different regions.

The income-generating effect of digital agriculture has been verified at multiple levels. Does the smartphone, as the main vehicle of digital agriculture, have the same income-generating effects for all farmers? In this paper, we construct four mechanisms through which smartphone use can impact farmers’ income in the context of digital agriculture. The specific path is shown in Figure 1.

Firstly, the use of new smartphone farming tools can reduce the cost of information searches for farmers and alleviate the information constraints that farmers suffer from [23]. It can also promote the growth of farmers’ income. In the Internet era, the price of agricultural products and market demand is relatively transparent, and buyers and sellers trade directly, eliminating intermediate links and preventing exploitation by mediators [24]. The use of new farming tools on smartphones has been a great help to farmers. The use of new smartphone farming tools plays an important role in breaking the low level of equilibrium, mitigating the information dilemma, and enhancing farmers’ ability to access information. According to search theory, farmers will reduce their search behavior when the cost of searching for information is higher, all other things being equal [25]. When the cost of searching for information is low, farmers will expand the scope and depth of their search and are more likely to obtain relevant, actionable information, thus optimizing their allocation of resources and increasing their income levels.

Secondly, the use of new smartphone farming tools promotes the transformation of agricultural chains and the diversification of trading markets [26]. The transparency of market information has changed the passive position of farmers in the supply chain. Farmers can take the initiative to introduce their agricultural products to the market through convenient e-commerce channels, thus changing the traditional model of low-value-added and low-profit supply, improving their marketing and services, and promoting the transformation of the agricultural industry chain. The use of smartphones can help small and medium-sized farmers sense market changes in a timely manner [27], expand their market reach to provide better access to domestic and international sales markets, and promote significant growth in sales territories. The expansion of markets, in turn, increases farmers’ opportunities to connect to larger markets, where they will also have access to more up-to-date information, thus creating a virtuous circle. The expansion of distribution channels and market reach provides more profitable opportunities for farmers.

Third, new smartphone farming tools enhance farmers’ access to information and knowledge [28]. The wider range of resources and information available to farmers using smartphones can strengthen their learning capacity and technical skills, which is conducive to enhancing farmers’ skilled human capital [29]. In addition, the use of smartphones by farmers to search for relevant health information has increased their focus on exercise and health care, contributing to the accumulation of health-based human capital. The internet also helps users acquire knowledge from traditional educational channels and promotes rural household entrepreneurship [30]. These are important avenues of human capital accumulation for farm households. The increase in human capital in farming households can increase their income levels.

Fourthly, from a social perspective, farmers’ use of smartphones as a new farming tool for social activities has significantly widened their social space. Farmers interact socially through smartphones, and the ease of access to knowledge and new agricultural skills leads to broader opportunities for farmers to increase their income [31]. These opportunities resonate throughout the agricultural value chain, thereby improving farmers’ livelihoods. They help reduce social exclusion, enhance social interactions between users, and enrich users’ circles of friends and networks, thus contributing to the accumulation of social capital and, ultimately, increasing the likelihood of increased income for farmers.

## 3. Research Hypothesis

The use of Internet technology by farmers has improved the ease of information transmission, reduced the threshold for farmers to access market information, agricultural technology, etc., and, to some extent, increased farmers’ awareness [32]. The Internet can effectively weaken the barriers to market information in time and space, effectively save costs, distribute the dividends of market expansion among farmers, and achieve increased income for farmers [33]. The rapid dissemination of agricultural and non-agricultural information brought about by the use of Internet technology has helped promote part-time employment. It has increased non-agricultural entrepreneurship and employment opportunities for farmers, ultimately contributing to an average increase of 25–30% in individual additional wage income [34]. This is the case with smartphones. As smartphones are new farming tools with wide coverage and strong penetration, the resulting market expansion dividend and speed of information dissemination will be even stronger, so the use of new smartphone farming tools by farmers will increase their income.

Therefore, the first hypothesis of this paper is the following.

**H1.**  *The use of new farming tools on smartphones will have an income-generating positive effect on farmers.*

There are differences in farmers’ returns due to the use of new farming tools on smartphones. This specifically reflects the uneven distribution of perceived returns due to the use of new smartphone farming tools. As individual farmers have very different resource endowments, the ability to fully recognize and apply new smartphone farming tools is key to their effective conversion and return. Some studies have found that farmers from different rural areas differ in their ability to apply and appreciate new farming tools, leading to differences in returns [35]. This return differential has led to a greater positive marginal effect of smartphone use among low-income people as the internet has become more widespread [36]. Some studies have even directly pointed out that the impact of internet use on smallholder technology adoption is greater for farmers with low levels of education, little training experience, and low incomes [37]. Therefore, the second hypothesis of this paper is as follows.

**H2.**  *The use of new farming tools on smartphones will have different income-generating effects for different income groups.*

The China Digital Economy Development Report 2020 shows that China’s digital economy has been distributed in a stepped pattern, with the scale of development gradually decreasing from the southeast coast to the western inland. This indicates clear digital divides between the east, middle, and west of China. Therefore, some scholars believe that the digital divide between economically developed and economically backward regions, and between small farmers and new large-scale business entities, will become increasingly wide [38].

Accordingly, the third hypothesis in this paper is the following.

**H3.**  *The use of new smartphone farming tools will have a different income income-generating effect on farmers in different regions.*

## 4. Data, Variables, and Model Construction

### 4.1. Data Sources and Basic Information of the Sample

This study uses microdata from the 2018 China Household Tracking Survey (CFPS). This data provides a detailed survey of the core variable of interest in this paper, namely the extent of use of new farming tools on smartphones (http://www.isss.pku.edu.cn/cfps/ (accessed on 10 December 2022)). Moreover, it covers data at the community, household, and individual levels, so this is a suitable, reliable source of data for this paper’s research questions. In this paper, the sample data was filtered by (1) screening the sample in “rural” areas, (2) eliminating those who did not use smartphones, and (3) eliminating those with null values and significant bias. This process yielded a valid sample size of 3019, covering 25 provinces: Tianjin, Hebei, Liaoning, Shanghai, Jiangsu, Zhejiang, Fujian, Shandong, Guangdong, Shanxi, Jilin, Heilongjiang, Anhui, Jiangxi, Henan, Hubei, Hunan, Sichuan, Chongqing, Guizhou, Yunnan, Shaanxi, Gansu, Guangxi, and Inner Mongolia.

### 4.2. Variable Setting

The explanatory variable is annual net household income. This paper measures the household property income of farm households by using the item “annual net total household income” in the CFPS questionnaire. In order to eliminate the adverse effects of heteroskedasticity, the property income of farm households is logged and included in the regression equation for this study.

The explanatory variable is the degree of use of new smartphone farming tools. Based on the theoretical analysis presented in the previous section, this paper starts with the implementation scenarios of new smartphone farming tools; the extent to which farmers use the new smartphone tools was further measured with the help of eight items in the questionnaire. Among them, “monthly smartphone bill” and “spare time for internet access” can be understood as measuring the basic level of new smartphone farming tools use [39]. The “frequency of using the Internet for study” and “frequency of using the Internet for work” could be said to measure the work and study dimension of smartphones [40]. The “frequency of using the Internet for entertainment” and “frequency of using the Internet for business” can be understood as reflecting the basic level of mobile phones and new farm tools’ digital life [41]. The “frequency of using the Internet for social interaction” and “frequency of sending and receiving emails” can be understood as reflecting the social aspect of the new smartphone farming tools [42]. The final calculation of the individual level of use of new smartphone farming tools is based on the proportions of different levels of use of new smartphone farming tools by individual farmers, with equal weighting. The main variables and descriptive statistics of this paper are shown in Table 1.

The control variables are based on previous studies. At the micro level, human capital, social capital, and household characteristics are included. At the macro level, the sample area is divided to minimize the regression bias caused by other variables in the new smartphone farming tools. At the human capital level, age, the highest level of education, and physical fitness are included. At the social capital characteristics level, “human gift expenditure” and “human relations” are included. At the level of household characteristics, “land assets”, “number of people eating at home”, and “cultural and recreational expenditure” are included.

### 4.3. Measurement Model Construction

In this paper, using the least squares (OLS) method, the income equation is as follows:

*lnY_i_* = *β*_0_ + *α*_1_
*X_i_* + *μ_i_*, (1)

where *lnY_i_* denotes the logarithm of the net income of the *i*th farm household; *α*_1_ denotes the use of new smartphone effect of farming tools; *μ_i_* denotes the random error term; and *X_i_* represents the human capital, social capital, household characteristics, and regional differences variables that can be observed in the *i*th farm household affecting the net income of the farm household per capita, including age, health status, the highest education level, expenditure on human courtesy, human relations, cultural and recreational expenditure, the number of people eating at home, land assets, and regional differences.

## 5. Empirical Tests and Analysis of Results

### 5.1. Baseline Model Estimation Results

This study uses ordinary least squares regression to estimate the impact of new smartphone farming tools on farm household income. The results, presented in Table 2, show that most of the *p*-values are within the acceptable range, showing that the method used in this paper is feasible.

The results indicate that the degree of use of new smartphone farming tools by farmers had a positive and significant effect on net household incomes at the 1% significance level. This means that the higher the level of use of new smartphone tools by farmers, the higher the household income. H1 is validated.

Farm household net income is also significantly impacted by farm household age, education level, physical condition, human courtesy, cultural and recreational expenditure, the number of people eating at home, land assets, human capital, social capital, and household characteristics.

### 5.2. Analysis of Endogeneity Issues

The extent of farmers’ use of new smartphone farming tools is not a random behavior or the result of random allocation. Rather, it is a choice made by farmers according to their own resource endowments, resulting in individual autonomy. Therefore, using OLS alone to estimate the impact of new smartphone farming tools on farmers’ income gives rise to the problem of bias due to self-selection. Moreover, farmers’ use of new smartphone farming tools may be determined by human capital, social capital, or household characteristics, which also impact farm household income. Thus, there is an endogeneity problem in estimating the impact of the degree of new smartphone farming tools use on farm household income, i.e., the degree of new smartphone farming tools used by farmers is related to farm household income and the error term.

Therefore, this study draws on previous research and selects “the mean of whether or not farmers in the village other than the farmer use the Internet (instrumental variable)” [43]. Two-stage least squares were used for estimation. According to the “peer effect” theory [44], a peer’s decision to use new farming tools on a smartphone influences a farmer’s decision to use them but does not affect the farmer’s income level.

As shown in Table 3, the estimation results of the first stage of instrumental variables show that, in a village, the average value of new farm tools used by other farmers except the sample farmers will have a significant positive impact on the degree of new farm tools used by the sample farmers. This suggests that higher levels of new smartphone farming tool use at the village level in the sample area have a catalytic effect on individual farmers’ new smartphone farming tool use. Consistent with the results of the baseline regression, this further validates the proposition, per H1, that the degree of new smartphone farming tools used by farmers has an income-enhancing effect on them. The F-test values for the first-stage regressions were all greater than the empirical value of 10, contradicting the original hypothesis of the existence of weak instrumental variables. The Hausman test rejected the original hypothesis that the mean value of new smartphone farming tools used by farmers in the village other than this farmer was an exogenous variable, indicating that the use of the instrumental variable estimation method is reasonable.

The results of the second stage of estimation show that the effects of human capital, social capital, and household characteristics, as well as the interaction term between the core explanatory variables on the household income term are similar to the baseline regression in terms of direction and level of significance. This suggests that the previous findings still hold after accounting for the potential endogeneity problem in the model.

### 5.3. Analysis of Intermediary Effects

In order to identify the mechanisms of the impact of new smartphone farm tools on farm household income, this paper introduces the household communication network cost (communication) to represent the social network and conducts the mediation effect analysis accordingly. The results show that there is a significant positive effect of social capital on the use of new smartphone farming tools, a positive effect of actual use of new smartphone farming tools on farmers’ income, and a significant positive effect of social networks on farmers’ income. This indicates that social networks exert significant positive mediating effects.

The Bootstrap test was used to examine the mediating effect of social capital in the relationship between Internet use and income, with a sample size set at 1000. The results showed that the direct effect was 0.004, which was significant at the 1% statistical level; the indirect effect was 0.048, which was significant at the 1% statistical level; and the confidence interval for the indirect effect was (0.173, 0.239), which does not contain 0, indicating that social networks play a mediating role in the relationship between the degree of use of new smartphone farm tools and the income of farm households. Farmers improve their ability to communicate with friends and family and break down old social taboos and barriers through the high-frequency use of new farming tools on smartphones [45]. Thus, H1 is further confirmed.

### 5.4. Heterogeneity Analysis and Extension Studies

Heterogeneity analysis usually subdivides the sample into different groups according to a particular variable. As different levels of use of new farming tools on farmers’ smartphones may have different effects on farmers of varying income levels, this study uses a quantile regression model to further analyze the differential impacts of internet use on high-, middle-, and low-income farmers. The results are shown in Table 4.

**Figure 2:**
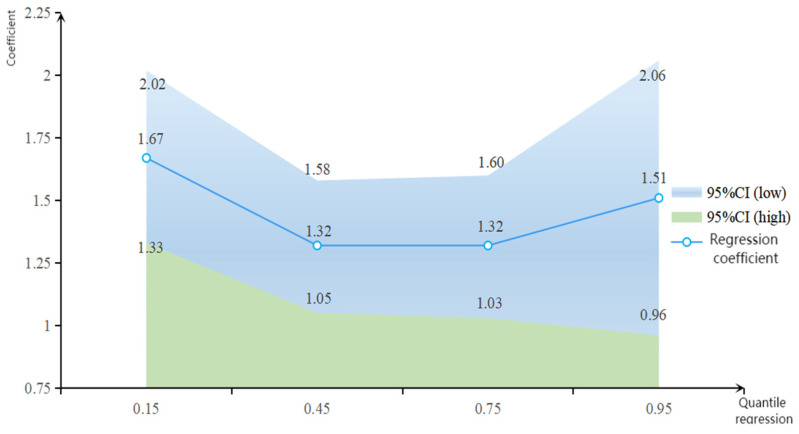
The mean of the quantile regression coefficients of the use of new smartphone farming tools on farm household income. Notes: (1) In this figure, the horizontal coordinate indicates the quantile, and the vertical coordinate indicates the coefficient. (2) The 95% CI is the 95% probability that the confidence interval includes the total when the sample is extrapolated to the total.

The results of different quantile coefficients are shown in Figure 2. The results of the regression of the income quantiles of farming households show that the overall coefficient of the regression of the quantiles of Internet use tends to fall and then rise as the quantiles rise. The coefficient on the degree of use of new smartphone tools decreases from 1.67 at 15% to 1.32 at 45% and then rises to 1.51 at 95%. Thus, Internet use has the greatest income-increasing effect on low-income farmers; it also has a strong income-increasing effect on high-income farmers; its income-increasing effect is lowest for middle-income farmers. Accordingly, H2 is verified.

To summarize, it can be seen that farmers’ use of new smartphone farming tools has a good income-generating effect. The possible reason for the highest income-generating effect on low-income farmers is that the use of new smartphone farming tools breaks down barriers to factor mobility, such as “information asymmetry” and “social barriers,“ thus reducing the gap between low-income farmers and high-income farmers. The marginal effect of the use of smartphone tools on the income of low-income farmers is higher than that of high-income farmers. High-income farmers use new smartphone farming tools to a greater extent, and their economic advantages give them sufficient payment capacity to bear the various costs arising from the use of new smartphone farming tools. So, their original advantages in human capital and social capital are further expanded, increasing their income. The lowest income-generating effect on middle-income farmers may be because the marginal dividend of the income-generating effect of their use of new smartphone farming tools decreases, and the income-generating effect brought about by the use of new smartphone farming tools is inefficiently transformed.

Regional differences are important determinants of disparities in farm household incomes. The degree of economic development varies among China’s regions, and factors such as economic base, resource endowment, and education level also make certain regional differences in levels of mobile internet development and the penetration of information technology in each region. Previous research found that China’s level of informatization development has obviously spatially uneven characteristics, with the eastern coastal region having the highest level of digitization and the southwest and northwest regions lagging behind [46]. To further analyze the regional differences in the extent to which the use of new smartphone farming tools affects the income gap of farming households, this paper further subdivides the 25 provinces into the eastern, central, and western regions. It uses group regression to divide the sample into three regional groups. The results are shown in Table 5.

The regression results of the subgroups show that farmers in the eastern, central, and western regions all exhibit income-generating effects from the use of new smartphone tools, with the western region having the highest: a positive and significant coefficient of 1.659 at the 1% level. The lowest income-generating effect was found in the central region, but it was also positive and significant at the 1% level, with a coefficient of 1.018. This validates H3.

Rural households in western China have the best income increase effect by using new farm tools with mobile phones. The reason is that the popularity of Internet services and digital products in remote and backward areas with an imperfect mobile Internet foundation is lower than that in urban areas, leading to a more serious information suppression phenomenon. The use of new smartphone farming tools has bridged the shortcomings of the consumer environment and services in the west, giving local residents direct access to a diverse range of agricultural information with transparent product pricing. It has also reshaped the deep-rooted consumer philosophy of farmers in the western region, making them more receptive to the consumption of digital products and information services and helping them to break down information barriers to a greater extent. In areas with lower levels of information infrastructure construction, there is a lower level of information development and poorer internet resources. As a result, the marginal effect brought about by farmers’ use of new farming tools on smartphones is stronger.

Moreover, in addition to promoting income generation in the form of breaking down information barriers, enhancing accessibility, improving coverage, and having good marginal effects, the use of new smartphone farming tools by farmers in the western region has also captured changes in farmers’ information needs with technology and created new digital supplies through innovative products, services, and scenarios. In this way, a virtuous circle has formed. The reason for the good effect of new smartphone tools on increasing income in the eastern region is the higher level of digitalization in areas with faster internet development, better mobile communication infrastructure, and a stronger internet atmosphere. The strong Internet atmosphere is conducive to promoting farmers’ use of new smartphone farming tools and also gives farmers a deeper knowledge of new smartphone farming tools, which in turn results in farmers using new smartphone farming tools to increase their income more efficiently.

A further reason for the lower income-generating effect in the central regions compared to the eastern and western regions may be that these regions are still dominated by traditional industries, making it difficult for farmers to gain more returns through the use of new smartphone farming tools.

## 6. Conclusions

In the context of the digital economy becoming an important economic form in the new era of China [47], vigorously promoting the development of digital agriculture is not merely an important way to solve the “three rural issues”. It is also necessary for the promotion of high-quality agricultural development [48]. Compared with the previous studies on the effect of digital agriculture on income generation, this paper innovatively introduces new cell phone farming tools for discussion, and the following three conclusions were drawn.

Firstly, the use of new farming tools on smartphones can significantly enhance the income of farmers. Farmers learn to work through the use of new smartphone farming tools, and the sales chain promptly connects to the consumer market to reduce transaction costs and ultimately increase revenue [49].

Secondly, farmers in the western region exhibit the highest income-generating effect from the use of new smartphone farming tools, followed by the eastern region, while this effect is weakest in the central region. In contrast, the western region has the characteristic of “a small boat with a good head start,” and farmers are more able to integrate digital agricultural resources quickly to maximize their value in the current period. In contrast, digitalization in the central region has a “siphon effect” between urban and rural areas, resulting in a less income-increasing effect than in other regions.

Thirdly, further analysis reveals that the rate of return for higher-income farmers using new farm tools is slightly lower than for lower-income farmers. The reason for this is that low-income farmers are prone to higher marginal effects from the use of new smartphone farming tools. As low-income farmers have relatively low income expectations when using new smartphone tools, their use is conducive to a “positive tunnel effect” [50], which promotes the further use of new smartphone tools by farmers.

## 7. Policy Implications

Based on the above conclusions, this paper puts forward the following policy recommendations. First, as traditional agriculture continues to transform into digital agriculture, the construction of digital infrastructure in rural areas should be further improved, and the driving force of digital technology should be given full play [51]. In addition, internet providers should offer farmers more affordable mobile Internet devices and customized smartphone packets; this further reduces the access threshold and the use cost of new smartphone farm tools.

Secondly, there should be continued efforts to promote digital agriculture development policies in the western region to narrow regional development inequalities. Toward these ends, the government should carry out special digital agriculture construction projects for the western region that promote the rapid integration of digital agriculture resources. Finally, it should continue to take advantage of the good digital infrastructure conditions in the eastern region and share its experience to guide the development of digital agriculture in the central region.

Thirdly, focus on supporting farmers’ use of digital agriculture and expanding the marginal effects of digital agriculture for low-income people. Agricultural extension staff could be engaged in promoting digital agriculture so that more farmers who do not have direct access to it can understand its usefulness. Additionally, the government should provide support services that encourage farmers to efficiently use digital agriculture, such as building special websites, public SMS services, or WeChat public numbers, to provide farmers with relevant agricultural information directly.

Compared to previous studies, this article is based on microdata to reveal the income-increasing effect of digital agriculture, whose variables are concrete and real from the perspective of new agricultural tools on smartphones. Previous studies mainly interpret the economic effects of digital agriculture from a macro perspective. Therefore, the research perspective of this paper had certain innovation and actual leading meanings. However, these variables could not reflect the whole story of digital agriculture, while the mechanism of the income-increasing effect of digital agriculture should be further studied with the progress of technology. At present, the research group has carried out the latest field survey in the Poyang Lake area. In further research, the deficiencies of this study will be gradually remedied to support the development of digital agriculture and sustainable livelihoods for households.

## Figures and Tables

**Table 1:** Main variables and descriptive statistics.

Variables	Explanation of Variables	Average Value	Standard Deviation	Maximum Value	Minimum Value
*Net household income/year*	Continuous variables (logarithmic)	10.954	0.811	13.592	7.090
*Extent of use of new farming tools for smartphones*	Continuous variables (normalized)	0.333	0.126	1	0
*Human capital*					
*Age*	According to actual survey data (years)	39.04	11.43	74	14
*Level of health*	According to actual survey data	2.197	1.156	4	1
*Highest qualification*	According to actual survey data	1.86	1.179	6	0
*Social capital*					
*Human relations*	According to actual survey data (points)	7.086	1.835	10	1
*Expenditures on favors and gifts/year*	Continuous variables (logarithmic)	7.678	1.773	10.779	0
*Family characteristics*					
*Land assets*	Continuous variables (logarithmic)	9.858	1.85	14.524	0
*Number of people eating at home/year*	According to actual survey data (persons)	4.058	1.783	13	1
*Cultural and recreational expenditure/year*	Continuous variables (logarithmic)	1.574	2.616	9.903	0
*Regional differences*	1 = East; 2 = Central; 3 = West	2.172	0.837	3	1

**Table 2:** Household income regression results using OLS models.

Variables	OLS	OLS	OLS	2SLS
*Depth of use of new farming tools for smartphones*	2.062 *** (0.112)	1.476 *** (0.133)	1.481 *** (0.132)	6.894 *** (0.820)
*Age*		0.004 ** (0.001)	0.003 (0.001)	0.024 *** (0.004)
*Level of health*		0.037 *** (0.012)	0.033 *** (0.012)	0.033 ** (0.015)
*Highest qualification*		0.094 *** (0.013)	0.082 *** (0.012)	−0.102 *** (0.031)
*Expenditures on favors and gifts/year*		0.046 *** (0.008)	0.044 *** (0.008)	0.033 *** (0.100)
*Human relations*		0.008 (0.008)	0.010 (0.007)	−0.012 (0.010)
*Cultural and recreational expenditure/year*		0.038 *** (0.005)	0.040 *** (0.005)	0.015 ** (0.007)
*Meal expenses/year*		0.072 *** (0.009)	0.071 *** (0.008)	0.082 *** (0.010)
*Land assets*		0.076 *** (0.008)	0.081 *** (0.009)	0.067 *** (0.01)
*Regional differences*			−0.103 *** (0.016)	−0.107 *** (0.020)
R^2^	0.103	0.220	0.231	0.993

Note: (1) **, and *** denote significance at the 5%, and 1% levels, respectively, with standard errors in parentheses. (2) The results in the first column indicate that establishing a one-dimensional regression between the depth of use of new smartphone farming tools and farmers’ income can verify that new farming tools on smartphones have a positive income-increasing effect. The second column of the calculation results shows that the same conclusion can be obtained after controlling for human capital, social capital, and household characteristics variables. The third column of calculations shows that after controlling for human capital, social capital, household characteristics variables, and regional characteristics, the use of new farming tools on smartphones still has a good positive income-increasing effect. (3) To address the possible endogeneity issue, this paper cites ”the mean of whether or not farmers in the village other than the farmer use the Internet” as the instrumental variable and calculates it by the 2SLS (two-stage least squares) method, which shows that H1 still holds. (4) The greater the value of R^2^ (R-squared after), the increase of control variables, the more effective the selection of control variables is, and the better the fit of the model. After adding the instrumental variables, the R^2^ value increased to 0.993.

**Table 3:** Mechanistic analysis of the impacts of the use of new smartphone farming tools on farm household income.

Variables	Social Networks	Farmers’ Income
*Depth of use of new farming tools for smartphones*	0.844 *** (0.13)	1.302 *** (0.124)
*Social network expenditure*		0.206 *** (0.017)
Control variables	Control	Control
Constant term	3.612 *** (0.139)	7.809 *** (0.143)
*Region differences*	Control	Control
R^2^	0.148	0.257

Note: (1) *** denotes significance at the 1% levels, respectively, with standard errors in parentheses. (2) After controlling characteristics and region variables, the depth of use of new farming tools on smartphones has a significant positive effect on both social networks and farmers’ income. This indicates that social networks mediate the relationship between the degree of use of new smartphone farm tools and the income of farm households.

**Table 4:** Quantile regressions of new smartphone farm tool use on farm household income.

	Quantile 0.15	Quantile 0.45	Quantile 0.75	Quantile 0.95
*Depth of use of new farming tools for smartphones*	1.675 *** (0.175)	1.316 *** (0.134)	1.316 *** (0.146)	1.511 *** (0.282)
*Age*	−0.001 (0.002)	0.002 (0.001)	0.004 *** (0.002)	0.005 (0.003)
*Level of health*	0.045 *** (0.017)	0.016 (0.013)	0.011 (0.013)	−0.019 (0.022)
*Highest qualification*	0.088 *** (0.017)	0.102 *** (0.013)	0.074 *** (0.014)	−0.003 (0.025)
*Expenditures on favors and gifts/year*	0.071 *** (0.009)	0.046 *** (0.008)	0.034 *** (0.009)	0.015 (0.018)
*Human relations*	0.026 ** (0.01)	0.010 (0.008)	0.005 (0.008)	0.004 (0.016)
*Household entertainment expenditure*	0.031 *** (0.007)	0.026 *** (0.005)	0.042 *** (0.006)	0.076 *** (0.011)
*Meal expenses/year*	0.07 *** (0.011)	0.077 *** (0.008)	0.074 *** (0.009)	0.087 *** (0.018)
*Land assets*	0.131 *** (0.008)	0.083 *** (0.007)	0.062 *** (0.01)	0.05 *** (0.017)
*Region*	−0.116 *** (0.022)	−0.108 *** (0.017)	−0.079 *** (0.018)	−0.075 ** (0.031)
Sample size	3019	3019	3019	3019
R^2^	0.165	0.134	0.106	0.105

Note: (1) **, and *** denote significance at the 5%, and 1% levels, respectively, with standard errors in parentheses. (2) In this table, the quantiles from the lowest to the highest represent farm households with low to high income, respectively. The highest coefficient is found for the quantile equal to 0.15, which represents the highest income-increasing effect of low-income farmers. The lowest coefficients are found for quantiles equal to 0.45 and 0.75, which represent the poor income-increasing effect of middle-income farmers. The next highest coefficient is at the quantile equal to 0.95, which represents a better income-increasing effect for high-income farmers.

**Table 5:** Subgroup regressions of new smartphone farming tools use on farm household income.

Variables	East	Central	Western
*smartph*	1.556 *** (0.226)	1.018 *** (0.226)	1.659 *** (0.201)
*age*	0.02 (0.003)	0.001 (0.003)	0.079 (0.018)
*health*	0.027 (0.023)	0.012 (0.021)	0.047 *** (0.018)
*education*	0.057 ** (0.027)	0.082 *** (0.023)	0.003 *** (0.018)
*lnpresent*	0.020 (0.013)	0.043 *** (0.013)	0.060 *** (0.012)
*relationship*	0.016 (0.014)	0.013 (0.014)	0.008 (0.01)
*lnculture*	0.052 *** (0.01)	0.036 *** (0.009)	0.032 (0.008) ***
*lnland*	0.054 *** (0.012)	0.070 *** (0.013)	0.126 *** (0.013)
*food*	0.097 *** (0.015)	0.065 *** (0.013)	0.054 *** (0.011)
Sample size	843	814	1362
R^2^	0.216	0.184	0.258

Note: (1) **, and *** denote significance at the 5%, and 1% levels, respectively, with standard errors in parentheses. (2) The regressions were grouped into eastern, central, and western based on the regional distribution of the sample. The results show that farmers in all regions have a positive and significant effect on their income by using smartphone farming tools. According to the regression coefficients, they reflect that the best effect on income increase is in the west, followed by the center, and the lowest in the east.

## Data Availability

All data generated and analyzed during this study are included in this article.
